# Rice husk-originating silicon–graphite composites for advanced lithium ion battery anodes

**DOI:** 10.1186/s40580-017-0118-x

**Published:** 2017-09-19

**Authors:** Hye Jin Kim, Jin Hyeok Choi, Jang Wook Choi

**Affiliations:** 1 Graduate School of Energy, Environment, Water, and Sustainability (EEWS), Korea Advanced Institute of Science and Technology (KAIST), 291 Daehak-ro, Yuseong-gu, Daejeon, 34141 Republic of Korea; 2 School of Chemical and Biological Engineering and Institute of Chemical Processes, Seoul National University, 1 Gwanak-ro, Gwanak-gu, Seoul, 08826 Republic of Korea

**Keywords:** Lithium ion battery, Magnesio-milling reduction, Meso-porosity, Rice husk, Silicon anode

## Abstract

Rice husk is produced in a massive amount worldwide as a byproduct of rice cultivation. Rice husk contains approximately 20 wt% of mesoporous SiO_2_. We produce mesoporous silicon (Si) by reducing the rice husk-originating SiO_2_ using a magnesio-milling process. Taking advantage of meso-porosity and large available quantity, we apply rice husk-originating Si to lithium ion battery anodes in a composite form with commercial graphite. By varying the mass ratio between these two components, trade-off relation between specific capacity and cycle life was observed. A controllable pre-lithiation scheme was adopted to increase the initial Coulombic efficiency and energy density. The series of electrochemical results suggest that rice husk-originating Si–graphite composites are promising candidates for high capacity lithium ion battery anodes, with the prominent advantages in battery performance and scalability.

## Introduction

High capacity silicon (Si) anodes have received much attention from the battery community because their superior specific capacities can increase the energy densities of lithium ion batteries (LIBs) substantially [1–4]. Nonetheless, the cycle lives of Si anodes do not usually meet the commercial standards because of the immense volume change of Si during repeated charge–discharge cycles [5–7]. The volume change causes the pulverization of active material, delamination of the electrode, and unstable solid–electrolyte-interphase (SEI) layer.

In an effort to address the aforementioned issues related to the volume expansion, a variety of nanostructured Si materials have been introduced, as the nanometer dimensions can effectively release the stress built during the volume expansion of Si. Si nanomaterials along this direction include diverse morphologies, such as nanoparticles (Si NP) [8–12], nanowires [13–16], two-dimensional (2D) nanosheets [17–19], and etc. Although these nanostructured Si demonstrated improved cycling performance to great extents, they suffer from low tap densities and are more liable to unwanted surface reactions due to the large surface-to-volume ratios [20, 21]. Thus, it is desired to develop Si microparticles in which internal nanostructures are embedded. Meso- or microporous Si materials are well-aligned to this design consideration because their particle sizes are in the micrometer range while the wall thicknesses of pores are in the nanometer range.

In developing meso- or microporous Si materials, one critical aspect is scalability and batch-to-batch consistency in the synthesis. In most syntheses of Si nanomaterials, it is difficult to avoid a certain level of deviation in particle size and morphology. In fact, the generation of Si microparticles is nontrivial to completely exclude. In this regard, the magnesiothermic or magnesio-milling reduction of rice husk-originating SiO_2_ (RH-SiO_2_) is advantageous, as rice husks have highly consistent pore structures and are produced in massive quantity worldwide (96 million tons in 2016) [22, 23]. SiO_2_ occupies ~20 wt% of rice husk, and mesoporous SiO_2_ can be easily obtained by a thermal treatment (>600 °C) of rice husks. Whereas mesoporous Si originating from rice husk SiO_2_, namely Si_RH_, has been recently demonstrated as an excellent LIB anode material, exclusive use of Si_RH_ leaves a gap before immediate commercial adoption. Since inevitable volume expansion of Si_RH_ is still large in such a way that the electrode swelling and charge–discharge reversibility are not as controllable as those of current graphite counterparts. Si_RH_ also suffers from low electrical conductivity. These limitations suggest Si_RH_–graphite composite as a more viable solution for the very next generations of practical cells.

Based on the given motivation, herein, we provide a systematic investigation on Si_RH_–graphite composites as practical LIB anodes. By varying the mass ratio between Si_RH_ and graphite, we endeavored to find an optimal condition and also see the effect of Si_RH_ in terms of cycle life focusing on Coulombic efficiency (CE). Also, in order to overcome the relatively low initial Coulombic efficiency (ICE) of Si, a controllable pre-lithiation scheme was employed. The present study is expected to serve as a useful ground in developing high capacity LIB anodes integrating Si materials, with great potential towards commercialization from the viewpoints of volumetric energy density and resource scalability.

## Results and discussion

**Fig. 1:**
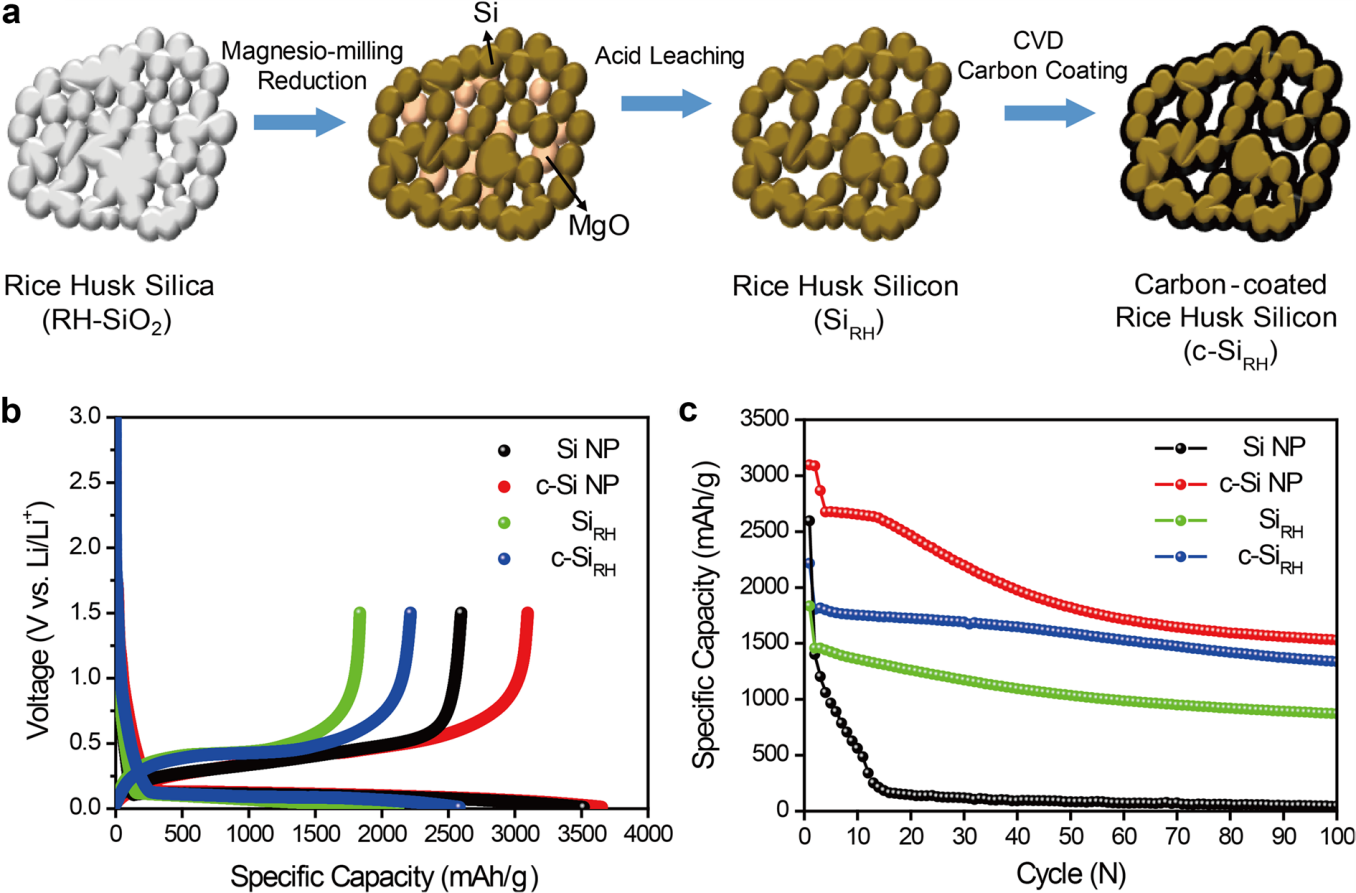
**a** Schematic illustration of synthesis of the carbon-coated rice husk silicon. Electrochemical measurements of various silicon electrodes. **b** Potential profiles in the first cycles at 50 mA/g and **c** cycling performance at 2000 mA/g of Si NP, c-Si NP, Si_RH_ and c-Si_RH_ electrodes

Si_RH_ was produced by a magnesio-milling process (Fig. 1a) by considering the scalability of the given process [24, 25]. In this synthetic scheme, Si and MgO were first generated after the magnesio-milling process. MgO was then removed by an acid treatment to yield bare Si_RH_. For electrochemical testing based on enhanced electrical conductivity, Si_RH_ was carbon-coated using a chemical vapor deposition (CVD) process. The carbon-coated Si_RH_ is denoted as c-Si_RH_.

Figure 1b displays the first charge–discharge profiles of four different Si electrodes: Si NP, c-Si NP, Si_RH_, and c-Si_RH_. Whereas all electrodes showed consistent lithiation and delithiation plateaus at around 0.1 and 0.4 V vs. Li/Li^+^, they delivered different gravimetric capacities. It is first noted that the carbon-coated derivatives exhibited larger specific capacities than those of the bare Si counterparts, indicating that the low conductivity of intrinsic Si is needed to be compensated for efficient Li storage. Also, both types of Si NPs showed clearly higher specific capacities than those of the Si_RH_ counterparts, which can be explained by the fact that the porous structure of Si_RH_ does not allow for as much volume expansion as Si NP, resulting in a smaller amount of Li storage in the given voltage range. The inevitable residue of silica in Si_RH_ is another reason for the smaller specific capacities. Considering the specific capacities of both Si_RH_ and c-Si_RH_ electrodes are far higher than those of typical commercial cathodes, the smaller specific capacities of Si_RH_ and c-Si_RH_ electrodes compared with those of the Si NP counterparts are unlikely to be a critical issue in practical cell design.

Figure 1c shows trade-off relation between specific capacity and cycle life: the higher the initial specific capacity, the more serious capacity drop during cycling. This phenomenon can be understood by the fact that the higher volume expansion of Si in realizing higher specific capacity causes the swelling of electrode and unstable SEI layer more seriously, leading to inferior cyclability. Detailed electrochemical data of the four electrodes are presented in Table 1.

**Table 1:** Electrochemical data of four different Si electrodes

	Reversible capacity (mAh/g)	ICE (%)	1st cycle (mAh/g)	100th cycle (mAh/g)	Retention (%)
Si NP	2596.3	62.4	1402.1	37.1	2.65
c-Si NP	3096.5	84.8	3087.5	1530.0	49.6
Si_RH_	1835.9	86.4	456.8	872.0	59.8
c-Si_RH_	2216.7	85.8	1803.5	1336.8	74.1

**Fig. 2:**
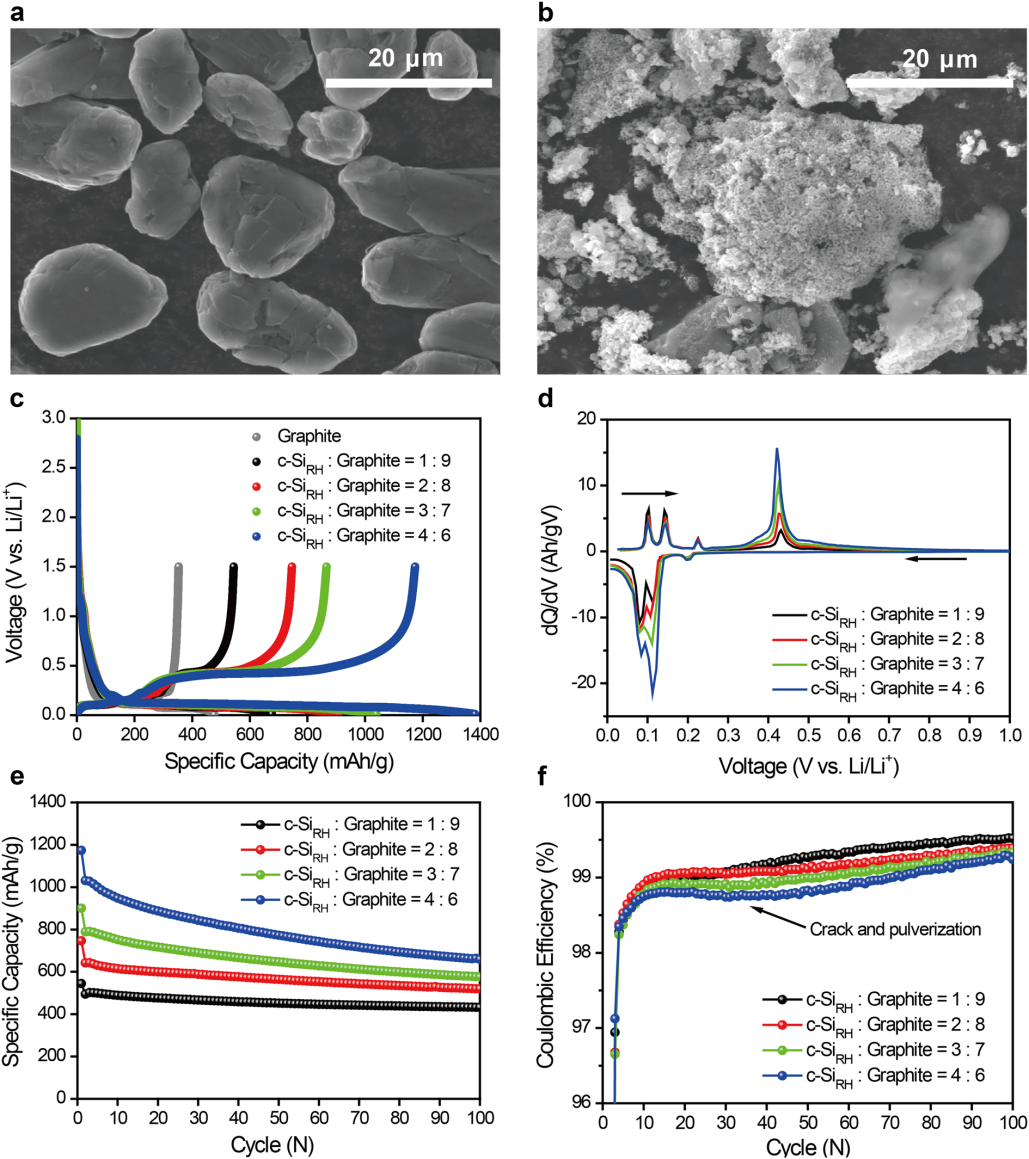
Characterization of c-Si_RH_ and graphite composites with different c-Si_RH_ and graphite mass ratios. SEM images of **a** graphite and **b** c-Si_RH_. **c** Galvanostatic charge–discharge profiles. **d** Differential capacity plots. **e** Cycling performance at a rate of 1C for 100 cycles (1C = 500, 700, 900 and 1100 mAh/g, respectively), and **f** corresponding Coulombic efficiencies

In order to see the commercial feasibility of c-Si_RH_, the electrochemical performance of c-Si_RH_–graphite composites with c-Si_RH_-to-graphite mass ratios from 1:9 to 4:6 were examined. The higher portions of graphite reflect its dominant occupation in current commercial cells. From morphology perspective, graphite showed smoother surfaces with more uniform particle sizes near 15 μm (Fig. 2a). In contrast, Si_RH_ forms secondary particles from primary particles (Fig. 2b) that correspond to the original rice husk powder.

As expected, the specific capacity increased with increasing c-Si_RH_ portion (Fig. 2c). The reversible capacity in the first cycle increased from 544.5 to 1173.6 mAh/g, as the composite ratio was varied from 1:9 to 4:6. The increased specific capacities of the composites with higher c-Si_RH_ portions were reflected in the longer plateaus at 0.1 V (lithiation) and 0.42 V (delithiation) in Fig. 2c and the enhanced peaks in differential capacity (dQ/dV) plots in Fig. 2d. In Fig. 2d, the two small peaks around 0.1 V assigned to the staging phenomenon of graphite decreased with decreasing the portion of graphite. The dQ/dV peaks also unveil the lithiation sequence in such a way that Li ions are first intercalated into graphite (stage IV and III) and then alloyed with Si. The ICE increased with increasing c-Si_RH_ portion, such as 80.5% of c-Si_RH_–graphite (1:9) to 84.9% of c-Si_RH_–graphite (4:6). This observation is attributed to the fact that the reactive edges of graphite are involved in the reaction with the electrolyte, although detailed mechanism needs to be unveiled via an additional in-depth study [26–28].

Similar to c-Si_RH_ alone, the composite electrodes exhibited trade-off relation between specific capacity and cycle life (Fig. 2e). While c-Si_RH_–graphite (4:6) started at 1030.6 mAh/g and ended at 657.5 mAh/g after 100 cycles, c-Si_RH_–graphite (1:9) started at 494.6 mAh/g and ended at 432.2 mAh/g after the same number of cycles. These capacity changes lead to capacity retentions of 63.8 and 87.4%, respectively. These results remind the challenge of Si_RH_, associated with the volume expansion of Si. The more challenging feature of higher portions of c-Si_RH_ with regard to cycle life was reflected in the CE data over cycling (Fig. 2f). The average CEs of c-Si_RH_–graphite (1:9) and c-Si_RH_–graphite (4:6) were 99.2 and 98.9%, respectively.

**Fig. 3:**
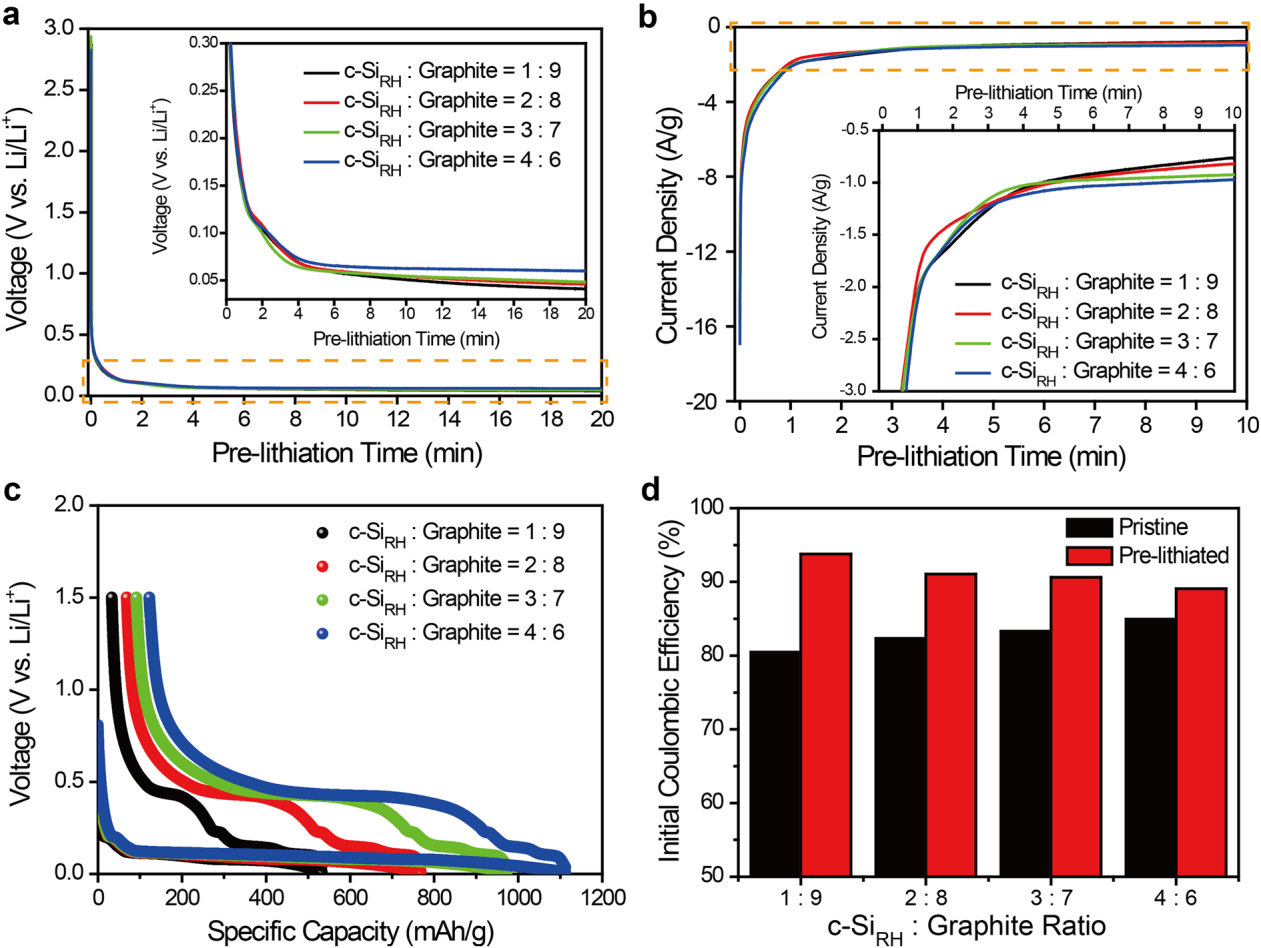
Characterization of pre-lithiation of c-Si_RH_ and graphite composite with different c-Si_RH_ and graphite mass ratios. **a** Voltage profiles during the pre-lithiation when 100Ω is integrated. **b** External short circuit current changes during pre-lithiation. **c** The first cycle voltage profiles after 6 min of pre-lithiation. **d** Comparison of the initial Coulombic efficiencies before and after pre-lithiation

It is known [3, 29] that increasing ICE is critical in practical cell design and the energy density of a full-cell. In an attempt to maximize the ICE, a pre-lithiation scheme was adopted [30, 31]. In particular, simple contact with Li metal foil while adjusting the circuit resistance was employed to control the degree and kinetics of lithiation more easily and accurately. Figure 3a displays the voltage profiles during pre-lithiation. With increasing the c-Si_RH_ portion, the voltage plateau during pre-lithiation becomes higher in reflection of the higher redox voltage of Si as compared with that of graphite. Since the given pre-lithiation uses the potential difference between both electrodes, a greater portion of graphite leads to lower cell potential and consequently to lower current density (Fig. 3b). This means that for the same pre-lithiation duration, the pre-lithiation of a higher portion of graphite may not be as efficient as that of a smaller portion of graphite. However, in practice, c-Si_RH_ plays a more dominant role in determining the ICE due to its high capacity, leading to smaller ICEs with increasing the c-Si_RH_ portions after pre-lithiation (Fig. 3c, d). The ICE decreased from 93.8 to 89.1% as the c-Si_RH_–graphite ratio was changed from 1:9 to 4:6. In all composite ratios, the ICE increased conspicuously to around 90%, reconfirming the usefulness of pre-lithiation. Notably, as different c-Si_RH_–graphite ratios yield the different levels of pre-lithiation, controllable pre-lithiation scheme is highly desirable in optimizing the cell conditions.

## Conclusions

In conclusion, we have tested c-Si_RH_ alone and c-Si_RH_–graphite composites with various compositions as LIB anodes, in order to take advantage of Si_RH_ in both structural and manufacturing aspects. While c-Si_RH_ demonstrated more sustainable operation than c-Si NP counterparts for long-term cycling, the trade-off relation between specific capacity and cycle life is to be overcome and optimized. The electrochemical performance of c-Si_RH_–graphite composites with various compositions exhibited a similar trade-off phenomenon, impairing the charge–discharge reversibility with increasing the c-Si_RH_ portion. Further enhancement in the reversibility is an important ‘must-solve’ task for these composites to be integrated into practical cells. Diminishing side reactions by advanced surface coatings and treatments may be a feasible solution along such direction.

## Experimental

### Synthesis of Si_RH_ via magnesio-milling

Rice husk powder was washed with 10% hydrochloric acid (HCl) solution to remove alkali metal impurities and dried at 70 °C for 5 h. The rice husk silica (RH-SiO_2_) was prepared by heat treatment of rice husk at 650 °C for 5 h under an air atmosphere using a furnace to remove organic components. To reduce the RH-SiO_2_ to Si_RH_, a magnesio-milling method was used. The stoichiometric amounts of RH-SiO_2_ (20 g) and magnesium (Mg) powder (16 g) were milled in an attrition-miller (KMAM-3C, KMTech) at 600 rpm.

Acid leaching was performed for the as-milled powder using 1 M HCl for 5 h at room temperature to selectively etching by-products such as MgO and Mg_2_Si.

To prepare the carbon coated samples (c-Si NP, c-Si_RH_), a chemical vapor deposition (CVD) process was used. Acetylene gas (C_2_H_2_) and Ar gas were flown into a furnace at 100 and 150 mL/min, respectively, for 5 min at 700 °C. After this coating process, carbon accounts for 6.6 wt% of the active material.

### Fabrication of electrodes

The active materials used in this study were graphite (POSCO Chemtech Co. Ltd), Si NP (Nanostructured and Amorphous Materials Inc., 50 nm) and Si_RH_. The morphology of the powder was examined by field-emission SEM (Nova230, FEI company).

For the electrode fabrication, a slurry was prepared by dispersing active material, polyacrylic acid (PAA, Aldrich, Mv ~ 3,000,000), and Super P (TIMCAL) in DI water at a mass ratio of 8:1:1. The slurry was then cast onto a copper foil and dried under vacuum at 70 °C for 12 h. The mass loading of the active material was 0.7 mg/cm^2^. Electrodes were punched into circular discs for fabrication of 2032 coin-type cells. The coin cells were prepared in an Ar-filled glove box by assembling a Celgard 2400 separator, the fabricated electrode (working electrode), and lithium metal foil (reference/counter electrode). A 1 M lithium hexa-fluorophosphate (LiPF_6_) solution in a mixture of ethylene carbonate (EC) and diethyl carbonate (DEC) (EC:DEC = 1:1, vol/vol) with 5 wt% fluoroethylene carbonate (FEC) (PANAX E-TEC) was used as an electrolyte.

To improve the ICE, pre-lithiation of the electrodes was employed. An external short circuit was constructed between the two electrodes (working and Li metal) and retained for 6 min. Within the external short circuit, a 100-Ω resistor was integrated to control the short circuit current. After pre-lithiation, the electrodes were assembled via the same procedure as described above.

### Electrochemical measurements

For battery testing, the galvanostatic mode was applied using a battery tester (PEBC05-0.01, PNE solution). The half-cell tests were conducted in a potential range of 0.01–1.5 V vs. Li/Li^+^. The first cycles were scanned at 100 mA/g under constant current (CC) mode for both charge and discharge and the subsequent cycles were scanned at 1C (1C = 2000 mAh/g for silicon electrodes and 1C = 500 mAh/g, 700, 900, 1100 mAh/g for 1:9, 2:9, 3:7, 4:6 composite electrodes, respectively.) under CC mode. During pre-lithiation, the short circuit current and voltage were monitored using a potentiostat (VMP3, Biologic).
